# Heterologous expression of ZjOMT from *Zoysia japonica* in *Escherichia coli* confers aluminum resistance through melatonin production

**DOI:** 10.1371/journal.pone.0196952

**Published:** 2018-05-07

**Authors:** Hongsong Luo, Chunyan He, Liebao Han

**Affiliations:** Turfgrass Research Institute, College of Forestry, Beijing Forestry University, Beijing, People’s Republic of China; Huazhong Agriculture University, CHINA

## Abstract

Melatonin is a molecule that can enhance the resistance of plants to abiotic stress. It can alleviate the damage of heavy metal ions, and other chemical substances, changes in temperature and humidity, oxidative stress in higher plants, and enhance resistance of plants to abiotic stress. The transformation of N-Acetyl-5-hydroxy tryptamin into melatonin requires the involvement of methyltransferase. In this study, a methyltransferase gene *ZjOMT* has been cloned from *Zoysia japonica*. The gene was induced by aluminum (Al) stress in the leaves and roots of *Zoysia japonica*, and was up-regulated by 20.86- and 31.18-folds, respectively. The expression of ZjOMT in *Escherichia coli* increased the content of melatonin by about 8-fold in the recombinant strain compared with that of the empty vector strain. Al resistance test showed that the resistance of recombinant strain BL21-pET32-ZjOMT to Al was significantly higher than that of the empty vector strain BL21-pET32. The survival rate of the recombinant strain expressing ZjOMT was about 100-fold higher than that of the empty vector strain when treated with 0.35 mM Al. These findings suggest that the heterologous expression of ZjOMT improved the resistance of *E*. *coli* to Al by increasing the content of melatonin.

## Introduction

Melatonin (N-acetyl-5-methoxytryptamine) is an important molecule that belongs to the class of indoles, and is widely used in animals and plants [1]. There were several studies on melatonin mainly in mammals. More in-depth research of melatonin in plants demonstrated its role in physiological regulation, and enhances plant resistance. It has been found that melatonin plays a key role in the defense system of *Arabidopsis thaliana* [2]. Melatonin can alleviate the damage of heavy metal ions, and other chemical substances, changes in temperature and humidity, oxidative stress in higher plants, and the provide resistance to plants in adverse environments [3,4,5]. For example, melatonin can reduce oxygen under high temperature stress in the production rate of cucumber and H_2_O_2_ content, effectively remove reactive oxygen species (ROS). It can also increase the inhibition of cell membrane permeability and MDA content, improve the antioxidant enzyme activity in cucumber leaves (such as SOD, POD, CAT) and the content of soluble protein. So the resistance of cucumber to high temperature stress is improved. Melatonin can also significantly reduce chlorophyll degradation of apple leaves under drought stress, improve leaf photosynthetic efficiency, alleviate oxidative damage, and effectively delay the leaf senescence [6]. Melatonin has the ability to resist ultraviolet stress in algae and higher plants [7]. It can also protect the integrity of the photosynthesis system and increase the chlorophyll content [8]. Exogenous melatonin treatment enhances the ability of plants to resist adversity, such as the significant reduction of germination rate of cucumber seeds under low temperature stress. Melatonin treatment can alleviate this phenomenon, improving the seed germination rate and seedling dry and fresh weight under stress conditions [9]. Exogenous melatonin can also relieve the cold stress of *Camellia sinensis* [10]. Exogenous addition of 1 μM melatonin effectively alleviates the inhibitory effects of salt stress on the growth of Hubei crabapple, by effectively alleviating chlorophyll degradation, increasing leaf photosynthetic rate and reducing oxidative damage [11,12]. Several studies have demonstrated that exogenous melatonin can significantly improve the survival rate of *pea* plant in high copper content soil. Studies reported that melatonin can improve the ability of plants to repair soil that is contaminated with heavy metals. The proper concentration of melatonin can effectively slow down Al induced toxicity in soybean [13].

The synthetic precursor of melatonin is tryptophan [14,15]. The production of melatonin from tryptophan involves a four-step continuous enzymatic reaction. Six enzymes are involved in this process including tryptophan decarboxylase (TDC), tryptophan hydroxylase (TPH), tryptamine 5-hydroxylase (T5H), serotonin N-acetyltransferase (SNAT), N-acetylserotonin methyltransferase (ASMT) and caffeic acid O-methyltransferase (COMT). The SNAT and ASMT/COMT are involved in the final two steps of melatonin synthesis [16]. The ASMT and COMT belong to O-methyltransferase family [17]. The COMT of *Arabidopsis* and rice also catalyzes the production of melatonin by the activity of ASMT [18,19]. Overexpression of MzASMT1, an apple ASMT in *Arabidopsis thaliana* increased the content of melatonin by 2–4 times [20]. Adverse stress conditions can promote the plant itself to synthesize melatonin. The expression of ASMT in rice could be induced by aging and some stress factors [17]. The melatonin contents in plants under different environmental conditions, especially in stress conditions such as drought, high temperature, oxidative stress, etc. demonstrated great differences [8,21]. The content of melatonin was increased significantly when *Aloe vera* was cultured at 25°C and then transferred to 4°C, and this suggested that melatonin might be involved in the resistance of plants under low temperature stress conditions [8]. High temperature may induce an increase in melatonin content in *Ulva*, and exogenous melatonin treatment could improve the resistance of macroalgae to high temperature stress [22]. Light has great effect on the content of endogenous melatonin in plants. The content of melatonin in roots and leaves of outdoor hyacinth that is planted under natural sunlight was 3 to 2.5 times that of the indoor light with relatively weak light intensity [23]. The concentration of melatonin in roots was significantly increased by 50μM Al treatment in soybean [13].

Zoysiagrass (*Zoysia japonica* Steud.) is commonly found in temperate climate regions and widely used for lawns [24]. Zoysiagrass is the most cold-tolerant grass among the warm-season turfgrasses and is widely cultivated in the world [25]. It has developed stolons and rhizomes, hard and thick subleathery leaves, and the lawn that was built has good elasticity, shear resistance, tolerance, salinity, high temperature and drought resistant properties. Zoysiagrass is also an Al resistant plant of turfgrass. It can also grow well in the acidic soil with high Al content in the south. It is more resistant to Al than many other grasses. We identified an annotated O-methyltransferase gene in the early stage of Al stress in Zoysiagrass transcriptome analysis, which was significantly up-regulated under Al stress conditions. In order to evaluate whether this gene plays a role in melatonin biosynthesis or it is helpful for Al resistance. The study of prokaryotic expression was performed. In this study, the gene was cloned, and a recombinant vector was constructed and expressed in *Escherichia coli*. The gene expression product and *E*. *coli* resistance were detected to explore the role of *ZjOMT* gene in melatonin synthesis and Al resistance and augment the research theory of melatonin synthesis for applying in the production practically.

## Materials and methods

### Plant materials and treatments

Using 400 μM AlCl_3_ solution to irrigate (each pot soil weighing 2 kg) and when the artificial climate indoor pot (at 28°C, 16 h light/ 8 h dark) was cultured for 1 year, *Zoysia japonica* varieties "Company" was grown up to 20 cm. Twenty plants per pot were planted and similarly three pots were repeated. The plants were treated for 10 consecutive days, and the roots and leaves of each basin were collected, respectively. Total RNA was extracted from leaf and root of Zoysiagrass using TRIzol reagent according to the manufacturer’s instructions (Tiangen, China). The first strand cDNA was synthesized using the Superscript™ III RNase H-Reverse Transcriptase kit (Invitrogen, USA).

### Isolation and sequence analysis of *ZjOMT*

Full-length cDNA sequence of *ZjOMT* was amplified using gene-specific primers ZjOMT-F: GGCACTGGGCCTGGACCAAGGATT and ZjOMT-R: CGCAGACTTAATATATACGAAGAAAC which were designed according to the *Zoysia* Genome Database (http://zoysia.kazusa.or.jp/). The PCR product was cloned into the pGEM-T (Promega, USA) and subsequently sequenced.

CDS was predicted by DNAMAN 7 software (Lynnon Corporation). The domain of the deduced ZjOMT protein was analyzed using Conserved Domain Database (CDD) (https://www.ncbi.nlm.nih.gov/cdd). The theoretical pI and molecular weight were predicted by the ProtParam online software (http://web.expasy.org/protparam).

### Expression pattern of ZjOMT in Al treated *Z*. *japonica*

The cDNA of Leaves and roots of the Al treated Zoysiagrass was used as template, q-ZjOMT-F: TGGTCGCTGATAGCCAAATC and q-ZjOMT-R: GCCGTCACCATTGATACCTT as primers. Zjactin was used as the reference gene. Primers were as follows: q-Zjactin-F: GTGCTTGACTCTGGTGATGGT q-Zjactin-R: GAACCACCAATCCAGACGCTG. qRT-PCR was performed on a Roche LightCycler 480 Real Time PCR System (Roche, Switzerland) in a final volume of 20 μl that contains 2 μl of a 1/10 diluted cDNA template, 10 μl of the 2× SYBR Premix Ex Taq (Takara, Japan) and 1.5 μl (5 mM) of gene-specific forward and reverse primers. Three biological replications and three parallel reactions were performed.

### Induction and expression of ZjOMT protein

Specific primers of additional enzyme cutting sites and 3 protective bases were designed according to the ZjOMT CDS sequences: ZjOMT-EcoRI-F: CCGGAATTCATGGCGCTTAGACTCTTAGCGGAAG (*Eco*R I restriction site underlined) and ZjOMT-HindIII-R: CCCAAGCTTTGGGTAGATCTCAATAACTGAAATGGG (*Hind* III restriction site underlined). PCR amplification was conducted using the following protocol: pre-denaturation for 5 min at 94°C, 30 cycles at 94°C for 30 s, 58°C for 30 s, 72°C for 30 s, followed by 1 cycle at 72°C for 10 min. Then PCR products and pET32a plasmids were used for double enzyme digestion using *Eco*R I and *Hind* III, respectively. The enzyme cut products were separated by agarose gel electrophoresis and after purification of the target fragment, T4 DNA ligase was used to connect the enzyme. The positive clones were obtained after transformation of the linked products. The recombinant plasmid of pET32-ZjOMT was obtained by PCR and sequencing. The recombinant plasmid was reformed using the *E*. *coli* BL21 DE3 PlysS expression strain. The pET32a transformed strains were used as control. Positive plaques were obtained after plaque detection by PCR and sequencing. BL21-pET32-ZjOMT recombinant strain and BL21-pET32a empty vector strain were cultivated by oscillating in 50 ml LB medium for 16 h at 37°C, respectively. 1 ml bacterial culture was transferred to 100 ml LB medium (including 0.2 mM IPTG), and then centrifuged to collect bacteria after 4 h. After ultrasonic breakage, SDS-PAGE gel electrophoresis was performed.

### N-acetyl-5-methoxytryptamine content test assay

BL21-pET32-ZjOMT recombinant strain and BL21-pET32a empty vector strain were cultivated in the LB medium by shaking culture at 37°C for 16 h, respectively. Then, 1 ml bacteria solution of the 2 strains were transferred to 10 ml fresh LB medium (1 mM IPTG), respectively. Induction and culturing was performed for 6 h, and then centrifuged at 8000 rpm for 5 min. 10μl supernatant samples were collected. Melatonin content was detected by the plant melatonin ELISA detection kit (R&D Systems, USA).

### Assay of Al stress tolerance of *E*. *coli* transformants

BL21-pET32-ZjOMT recombinant strain and BL21-pET32a empty vector strain were cultivated by oscillating in 50 ml LB medium at 37°C and cultured for 16 h. The LB medium was diluted by 10, 10^2^, 10^3^ and 10^4^ times, respectively. The bacterial solution of 5μl at different concentrations was added to solid 2×GM medium (with 0.2 mM IPTG) containing 0.4 mM AlCl3 and no Al(control),respectively. They were cultured at 37°C for 96 h. Then the growth of the colonies was observed.

Recombinant *E*. *coli* growth curve under Al stress: The 20μl of remaining bacterial solution of the above strains was taken, and inoculated into the 48 whole enzyme labelled plates containing 980μl LB liquid medium (including 350 μM AlCl_3_ and 0.2 mM IPTG). The growth curve was detected in the TECAN infinite M200 PRO enzyme labyrinth (TECAN, Austria) after sealing the membrane. The incubation temperature was set at 37°c, and the detection wavelength was 600 nm. The amplitude of the linear vibration was 6 mm, and the readings were taken every 1 h. Each strain was set up for 6 repetitions, and the growth curve was drawn after culturing for 18 h.

### Statistical analysis

All statistical analyses were performed using the Statistical Package for Social Sciences (SPSS 19.0) with Student’s t test in a two-tailed manner. Statistical significance was set at P < 0.01.

## Results

### Cloning and sequence analysis of *ZjOMT*

To investigate the molecular function of *ZjOMT*, we obtained its CDS with a length of 1080 bp (Table 1). This gene encodes a protein containing 359 amino acids with a predicted theoretical isoelectric point and molecular weight of 5.52 and 39.026 kDa, respectively.

**Table 1:** Nucleic acid and amino acid sequences of ZjOMTPosition.

	Sequence																			
1	atg	gcg	ctt	aga	ctc	tta	gcg	gaa	gtg	agt	cca	cag	gac	ttg	ctt	gta	gct	ctt	acc	gag
1	M	A	L	R	L	L	A	E	V	S	P	Q	D	L	L	V	A	L	T	E
61	ttt	cac	aac	cac	atg	ata	ggt	tat	gtc	aag	tca	atg	gcc	ctc	aag	tgc	gcc	gtg	gat	ctt
21	F	H	N	H	M	I	G	Y	V	K	S	M	A	L	K	C	A	V	D	L
121	ggc	atc	ccc	gac	gtt	atc	cac	cgc	cgc	ggc	ggt	gca	gcc	acc	ctt	gct	gac	att	gta	act
41	G	I	P	D	V	I	H	R	R	G	G	A	A	T	L	A	D	I	V	T
181	gac	act	gcg	gtg	cat	cca	gcc	aag	atc	tcg	gac	ctc	cag	cgc	gtg	atg	gag	ctg	ctt	agc
61	D	T	A	V	H	P	A	K	I	S	D	L	Q	R	V	M	E	L	L	S
241	tct	tca	ggt	atg	ttc	agt	act	ggg	gaa	gac	agc	aac	ggt	gct	gtc	atg	tac	agg	tta	aca
81	S	S	G	M	F	S	T	G	E	D	S	N	G	A	V	M	Y	R	L	T
301	act	ccg	ggc	cgc	ttc	tta	gtg	ggc	gag	cgc	aat	ctc	tct	ccc	atg	gta	ccg	ttc	ctg	gtg
101	T	P	G	R	F	L	V	G	E	R	N	L	S	P	M	V	P	F	L	V
361	agc	cct	ctc	gtc	gtc	tcc	tca	ttc	ttc	agc	atg	agc	gac	tgg	ctt	agg	tgc	gag	ccg	gcg
121	S	P	L	V	V	S	S	F	F	S	M	S	D	W	L	R	C	E	P	A
421	gtc	agc	ggc	tct	cta	ttt	gag	ctg	tca	cat	ggc	tgc	cca	cag	tgg	gag	atg	gcg	agc	aag
141	V	S	G	S	L	F	E	L	S	H	G	C	P	Q	W	E	M	A	S	K
481	gat	gcc	acg	ttc	aat	aga	ata	ctg	aat	ggc	tcc	atg	gtc	gct	gat	agc	caa	atc	ttc	ctt
161	D	A	T	F	N	R	I	L	N	G	S	M	V	A	D	S	Q	I	F	L
541	gaa	gtc	gtc	att	cta	gac	aag	ggc	cat	atc	ttc	cgc	ggg	ttg	aag	tca	ctg	gtt	gat	gtg
181	E	V	V	I	L	D	K	G	H	I	F	R	G	L	K	S	L	V	D	V
601	ggt	gga	ggc	cgc	ggc	gcg	gct	gcg	cag	gtc	ctc	gcg	tcc	tcg	ttt	cca	cgc	atc	aag	tgc
201	G	G	G	R	G	A	A	A	Q	V	L	A	S	S	F	P	R	I	K	C
661	act	gtc	cta	gac	ctt	ccc	cat	gtg	att	aat	caa	ggt	atc	aat	ggt	gac	ggc	aat	ctg	caa
221	T	V	L	D	L	P	H	V	I	N	Q	G	I	N	G	D	G	N	L	Q
721	ttc	gtc	gct	ggc	gac	atg	ttt	gag	tcc	att	cca	cct	gct	gat	gcc	gtc	tta	ctc	aag	aat
241	F	V	A	G	D	M	F	E	S	I	P	P	A	D	A	V	L	L	K	N
781	att	ttg	cat	gac	tgg	gct	gat	gat	gat	tgc	atc	aag	att	cta	aaa	cgt	tgc	aag	gaa	gca
261	I	L	H	D	W	A	D	D	D	C	I	K	I	L	K	R	C	K	E	A
841	atc	cct	gct	aga	aat	gat	ggg	gga	aaa	gtg	ata	atc	ata	gat	atg	gtg	aga	ggg	tcg	ctc
281	I	P	A	R	N	D	G	G	K	V	I	I	I	D	M	V	R	G	S	L
901	cag	gga	aac	aca	aaa	atc	agt	gag	atg	gaa	gcc	tca	cag	aac	ctg	ttc	atg	atg	tcc	atc
301	Q	G	N	T	K	I	S	E	M	E	A	S	Q	N	L	F	M	M	S	I
961	aat	ggg	gtg	gaa	cga	ggg	gaa	agt	gaa	tgg	aag	aag	tta	ttt	tcc	gct	gca	gga	ttc	agt
321	N	G	V	E	R	G	E	S	E	W	K	K	L	F	S	A	A	G	F	S
1021	gac	aac	tac	aag	atc	atg	caa	ata	ttg	ggt	ccc	att	tca	gtt	att	gag	atc	tac	cca	taa
341	D	N	Y	K	I	M	Q	I	L	G	P	I	S	V	I	E	I	Y	P	*

*Nucleic acid sequence is consisting of triplet lowercase letters, while amino sequence is consisting of single uppercase letters.

**Fig 1:**
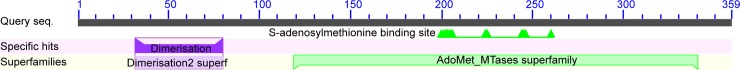
Conserved domains of ZjOMT.

The ZjOMT amino acid sequence was used to search and analyze the conserved domains in CDD. The gene contains dimerization domain and S-adenosyl methionine binding site that belongs to Ado-Met methyltransferase (Fig 1).

### Expression analysis of *ZjOMT* in Al treated *Z*. *japonica*

**Fig 2:**
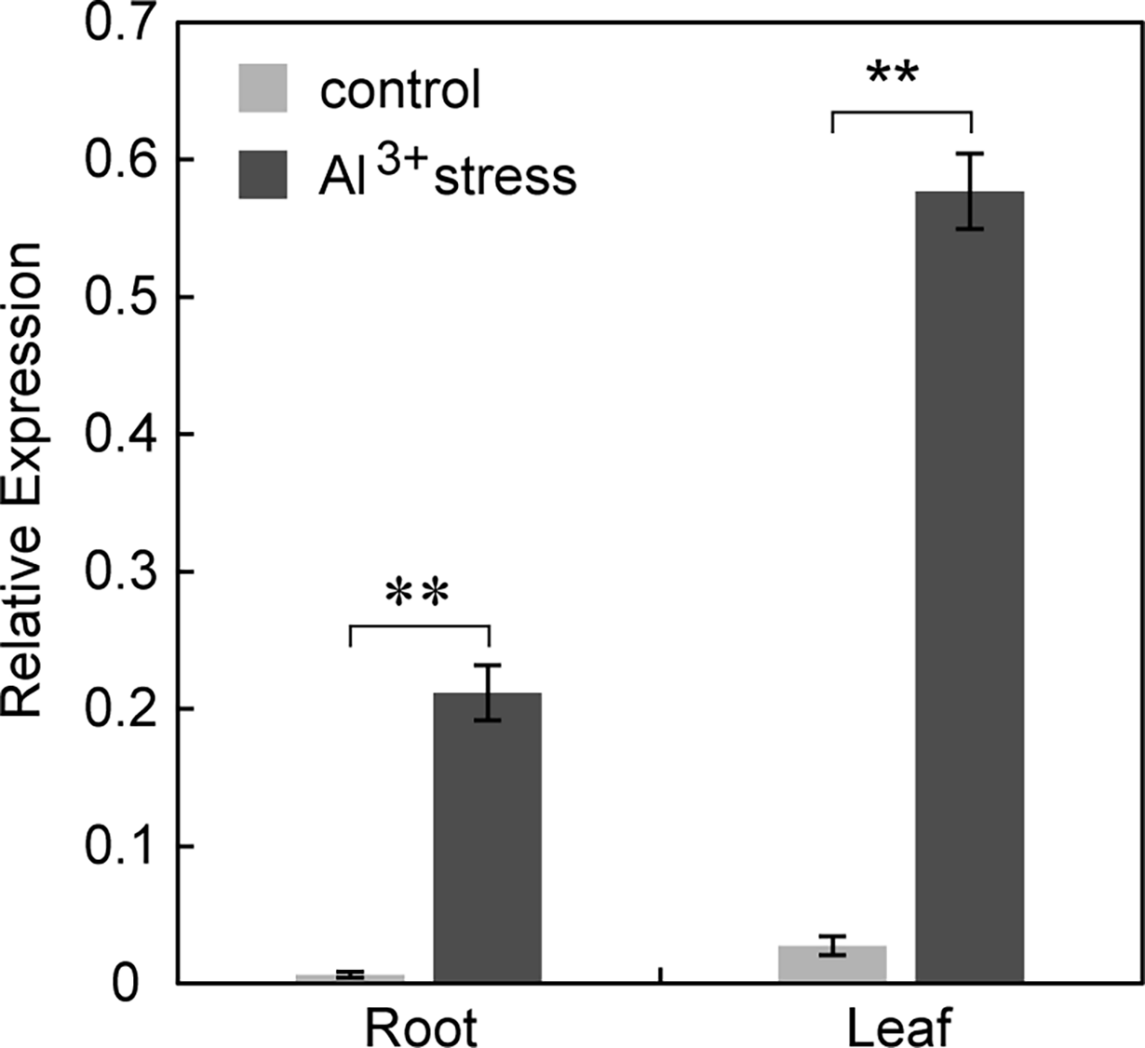
Expression of ZjOMT in Al treated *Z*. *japonica*. Light grey indicated without Al control, while the dark grey indicated 400 μM Al stress. “**” indicated the difference of P<0.01 in t test.

The expression of *ZjOMT* in *Zoysia* leaf was about 3-folds higher than that in root. After AlCl_3_ stress conditions, at 400 μM per kg of soil was treated for 10 d. The expression of *ZjOMT* in leaves and roots of Zoysiagrass was induced by Al stress and increased by 20.86-fold and 31.18-fold, respectively (Fig 2). The difference is significant (P<0.01).

### Induction of fusion protein in recombinant *E*. *coli* cells

**Fig 3:**
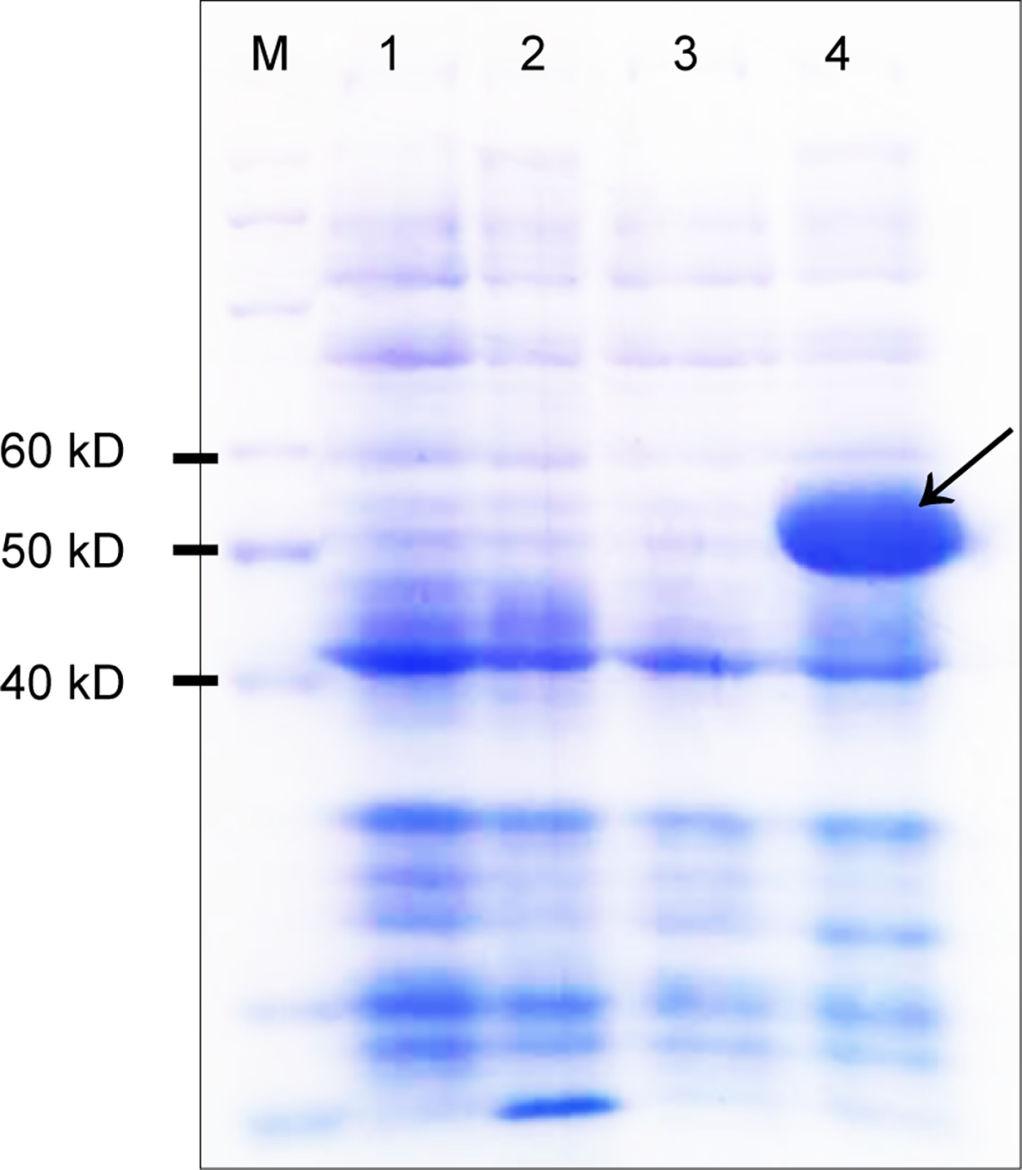
Total protein by SDS-PAGE electrophoresis of BL21-pET32a empty vector strain and BL21-pET32-ZjOMT recombinant strain. Lane 1 showed whole-cell proteins of uninduced empty vector strain of *E*. *coli* BL21-pET32a; Lane 2 showed whole-cell proteins of induced empty vector strain of *E*. *coli* BL21-pET32a; Lane 3 showed whole-cell proteins of uninduced recombinant strain *E*. *coli* BL21-pET32-ZjOMT1; Lane 4 showed whole-cell proteins of induced recombinant strain *E*. *coli* BL21-pET32-ZjOMT1; Lane M is protein marker.

The coding region of *ZjOMT* gene was amplified with two primers, designed and cloned into the pET-32a(+) plasmid to construct pET32-ZjOMT expression vector to analyze the expression of fusion protein. Results showed that the ZjOMT protein was expressed in Bl21 DE3 PlysS *E*. *coli* strain. The total protein of recombinant strains was analyzed by SDS-PAGE. A band of about 58 kD fusion protein was clearly observed in gels, indicating that this vector works normally and *ZjOMT* protein was expressed in the transformed *E*. *coli* BL21 cells after 3 h induction using 0.2 mM IPTG (Fig 3).

### ZjOMT overexpression enhanced N-acetyl-5-methoxytryptamine content of *E*. *coli*

**Fig 4:**
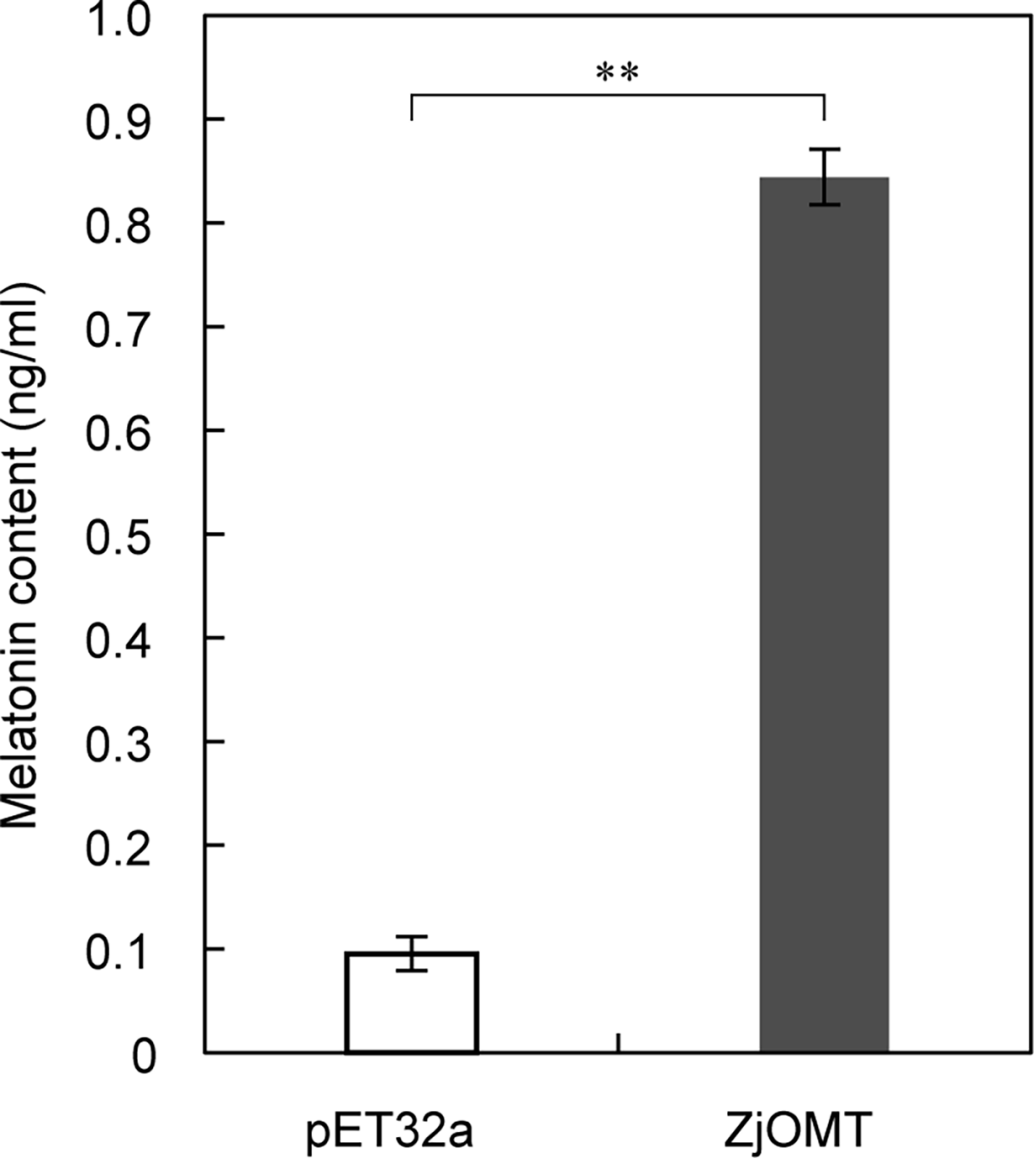
Production of melatonin by BL21-pET32-ZjOMT and BL21-pET32a empty vector strains induced by IPTG. The column chart was presented as mean±SD, white indicated an empty vector strain, the dark grey indicated ZjOMT recombinant strain, “**” indicated the difference of P<0.01 in t test.

The recombinant strain of BL21-pET32-ZjOMT was induced by 1 mm IPTG for 6 h. The content of melatonin in the bacterial liquid supernatant was significantly higher than that of the empty vector strain, and was about 8-fold that of the empty vector strain (Fig 4). This showed that the *ZjOMT* gene was involved in the production of melatonin.

### ZjOMT overexpression enhanced *E*. *coli* growth under Al stress

**Fig 5:**
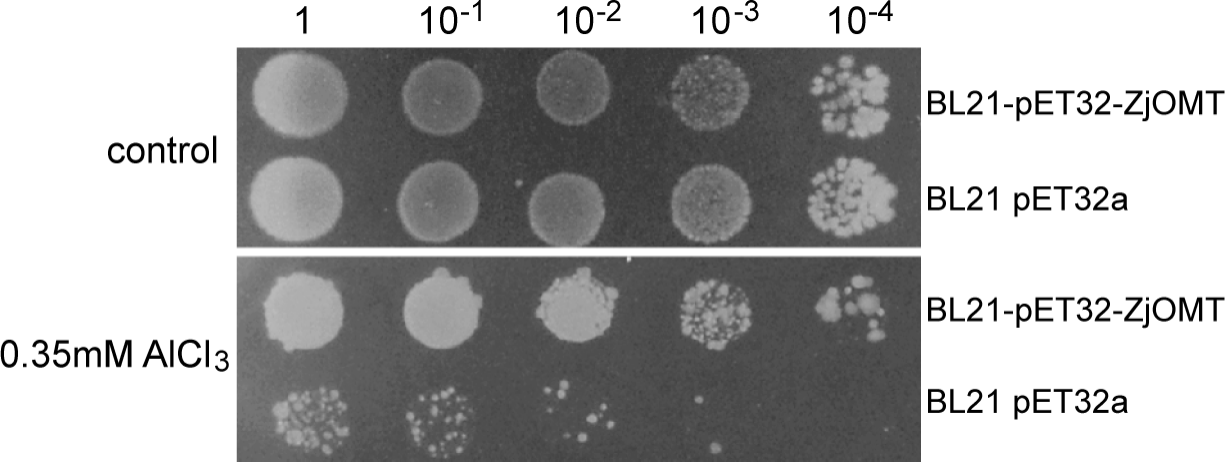
Growth of BL21 -pET32a and BL21-pET32-ZjOMT under Al stress.

To evaluate the effect of *ZjOMT* gene induced expression on host bacterial stress resistance, BL21-pET32-ZjOMT recombinant strain and BL21-pET32a empty vector *E*. *coli* were treated with Al stress. The growth of the empty vector strain was severely inhibited under Al stress, the survival rate of 100-times-diluted empty vector strain was similar to that of 10000-times-diluted recombinant strain, so the survival rate was about 1/100 of the recombinant strain (Fig 5).

**Fig 6:**
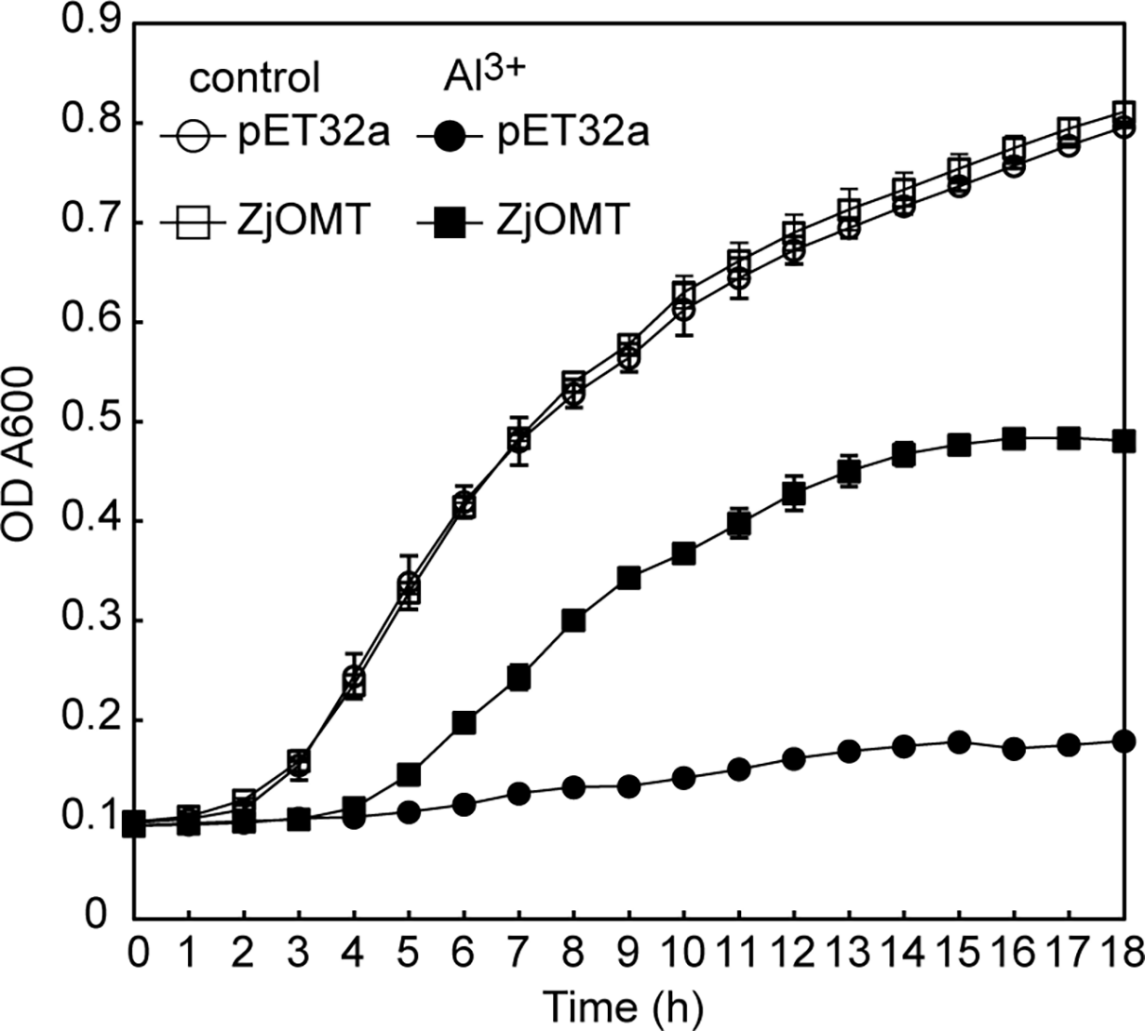
Growth curve of BL21 pET32a and BL21-pET32-ZjOMT under Al stress.

In the control medium without Al, the growth rate of BL21-pET32-ZjOMT recombinant strain and the empty vector strain remained similar. In the medium containing 350 μm AlCl_3_, the growth rate of the recombinant strain of BL21-pET32-ZjOMT was significantly higher than that of empty vector strain. The reproduction of the empty vector strain was obviously inhibited by Al, and the final concentration of the bacteria was about 1/3 that of the recombinant strain (Fig 6). These results indicated that the expression of *ZjOMT* gene significantly increased the resistance of *E*. *coli* to Al toxicity.

## Discussion

Melatonin plays an important role as antioxidant in plants [8,26,27]. Al stress produces oxygen for living. Antioxidation may be an important way for melatonin to resist Al. Melatonin in soybean reduces the toxicity of Al by regulating the antioxidant system [13]. Heterologous expression of ZjOMT in *E*. *coli* increased the content of melatonin and enhanced the resistance of *E*. *coli* to Al. Heterologous expression of ZjOMT may also reduce the toxicity of Al to *E*. *coli* by removing the ROS through melatonin. Therefore, ZjOMT may not only enhance the resistance of Al, but also increase the resistance to other factors such as drought, salt, heavy metal, cold and other adversities of reactive oxygen damage. For example, melatonin can improve the plants’ salt tolerance and promote its growth [28].

In addition to the antioxidative regulation of plant resistance, melatonin also regulates plant resistance by alleviating the expression of related genes. For example, endogenous melatonin of *Arabidopsis* can induce stress under high temperatures, resulting in the continuously increased content, while exogenous melatonin pretreatment can improve plant tolerance to high temperatures, where high temperature environment and the expression of exogenous melatonin induces the transcription factor HSFA1. This in turn activates the downstream gene, providing high tolerance to high temperatures in *Arabidopsis* [29]. Melatonin acts as not only a pineal hormone, but also an important signaling substance of functioning in animals [30], but also as a signaling substance in plants to regulate plant resistance. Melatonin acts as not only an antioxidant, but also regulates the resistance of plants by regulating the expression of other genes in the process of resistance.

The proper concentration of melatonin can effectively slow down the Al-induced phytotoxicity in soybean [13]. High melatonin content inhibited plant growth. Melatonin was used to treat the stem segments of *Polygonum cuspidatum* in vitro, and 3 μM and 6 μM melatonin demonstrated significant effects on the growth of the established stem segments of *Polygonum cuspidatum* and seedling growth of *Polygonum cuspidatum*. When the concentration was 6 μM, the height of the seedlings was increased by 87.5% and the leaf area was increased by 67.5%. However, when the concentration reached 24 μM, the growth of the seedlings was inhibited. The growth rate of *Scutellaria amoena* callus was more than 1 time higher than that of the control by 0.1 μM exogenous melatonin treatment, while 100.0 μM inhibited the proliferation of callus and differentiation of adventitious buds [31]. It was considered that the content of melatonin also has a dose effect in the resistance to Al stress of Zoysiagrass, and only an appropriate amount can better play the role of resistance. Further research on these points is warranted.

COMT is a multifunctional enzyme with ASMT activity. Rice COMT catalyzes the transformation of serotonin to melatonin, while rice ASMT1 showed no obvious conversion of serotonin to melatonin in *E*. *coli* [32]. There was a great difference between the types of catalytic substrates and the activity of enzymes in the O-methyltransferase family, and the ability to catalyze the production of melatonin was different. The expression of ZjOMT in *E*. *coli* significantly increased the production of melatonin, indicating that it has a considerable catalytic activity in the conversion of serotonin to melatonin.

## Conclusions

In this study, we showed that overexpression of ZjOMT increases the content of melatonin and the resistance to Al stress in *E*. *coli*. Therefore, our findings reveal that ZjOMT can be a useful target of genetic engineering for improving the tolerance of plants to Al stress.
